# Narrow Complex Ventricular Tachycardia

**DOI:** 10.7759/cureus.1423

**Published:** 2017-07-04

**Authors:** Murtaza Sundhu, Mehmet Yildiz, Sajjad Gul, Mubbasher Syed, Idrees Azher, Robert Mosteller

**Affiliations:** 1 Internal Medicine Residency, Fairview Hospital, Cleveland Clinic, USA; 2 Electrophysiology, Fairview Hospital, Cleveland Clinic, USA

**Keywords:** ventricular tachycardia, cardiac arrest, electrophysiology, myocardial infarction

## Abstract

Myocardial infarctions are frequently complicated by tachyarrhythmias, which commonly have wide QRS complexes (QRS duration > 120 milliseconds). Many published criteria exist to help differentiate between ventricular and supraventricular mechanisms. We present a case of a 61-year-old male with a history of hypertension, hyperlipidemia and coronary artery disease with prior stenting of the right coronary artery (RCA). He had been noncompliant with his antiplatelet medication and presented with cardiac arrest secondary to in-stent thrombosis. He was resuscitated and his RCA was re-stented, after which he made a good neurological recovery. During cardiac rehabilitation several weeks post-intervention, he was noted to have sustained tachycardia with associated nausea and lightheadedness, but no palpitation symptoms, chest pain or loss of consciousness. He was sent to the emergency department, where his electrocardiogram showed a tachycardia at 173 beats per minute which was regular, with a relatively narrow QRS duration (maximum of 115-120 msec in leads I and AVL) with a slurred QRS upstroke. This morphology was significantly different from his QRS complex during sinus rhythm. Intravenous diltiazem was ineffective but an amiodarone bolus terminated the tachycardia. The patient was admitted to the coronary care unit and treated with intravenous amiodarone infusion. A subsequent electrophysiology study was performed, showing inducibility of the clinical tachycardia. Atrioventricular (AV) dissociation was present during the induced arrhythmia, confirming the diagnosis of ventricular tachycardia. An implantable cardiac defibrillator was placed and the patient was discharged.

## Introduction

Arrhythmic complications of myocardial infarction (MI) may be divided into conduction abnormalities, bradyarrhythmias, and tachyarrhythmias. Conduction abnormalities include first-degree atrioventricular (AV) block, as well as second-degree and transient third-degree AV block, all common following acute inferior myocardial infarctions. Other conduction abnormalities include right and left bundle branch blocks, more common following acute anterior myocardial infarctions. Post-MI bradyarrhythmias include sinus bradycardia and various degrees of AV block. Post-MI tachyarrhythmias may be supraventricular (sinus tachycardia, atrial fibrillation, and paroxysmal supraventricular tachycardia in decreasing order of frequency) or ventricular (ventricular premature beats, ventricular tachycardia, and ventricular fibrillation). Post-MI tachycardia which has a QRS duration of < 120 msec are normally supraventricular in mechanism. Those with a QRS of > 120 msec may be ventricular or supraventricular tachycardia with aberrancy. Because narrow-complex tachycardia is nearly always supraventricular in mechanism, the possibility of a ventricular origin is generally not considered. We present a case of a patient presenting with narrow-complex tachycardia that was initially treated as supraventricular tachycardia, but later recognized and treated as ventricular tachycardia (VT). Informed consent statement was obtained for this study.

## Case presentation

**Figure 1:**
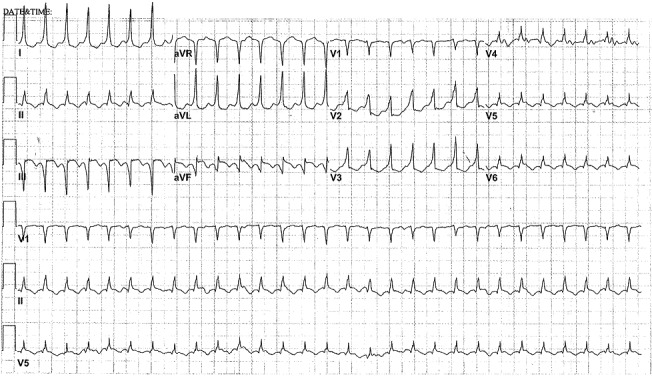
Electrocardiogram of the tachycardia

**Figure 2:**
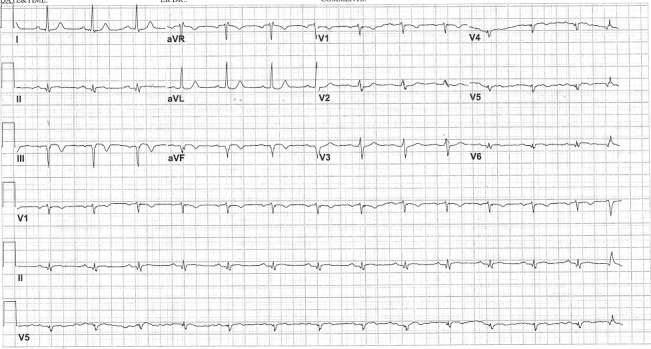
Electrocardiogram of the sinus rhythm

A 61-year-old male with a past medical history of hypertension, hyperlipidemia and coronary artery disease with the remote stent in the right coronary artery (RCA) presented with sustained tachycardia. He had suffered an acute ST-segment elevation myocardial infarction one month earlier and had undergone emergent RCA stenting with a drug eluting stent. Following that index admission, he had been discharged but presented three days later with a cardiac arrest, from which he was resuscitated. He had been noncompliant with his dual antiplatelet therapy and was found to have RCA in-stent thrombosis requiring repeat stenting with a drug eluting stent. After a good neurological recovery, he was again discharged and subsequently enrolled in cardiac rehabilitation. During the rehabilitation session, he was found to have sustained tachycardia with associated nausea and lightheadedness but no symptoms of palpitation, chest pain or dyspnea. He was treated with intravenous adenosine in the ambulance and allegedly had some brief sinus rhythm, but arrived in the emergency department in a regular tachycardia with hemodynamic stability. The electrocardiogram showed tachycardia at the rate of 176 beats per minute which was regular with narrow complexes in most leads and a maximum QRS duration of 115-120 milliseconds in leads I and aVL, with slurring of the QRS upstroke. There was an abrupt R-wave transition between leads V1 and V2 (as in Figure 1). The arrhythmia failed to respond to intravenous diltiazem but resolved after 150 mg of intravenous amiodarone. The sinus rhythm electrocardiogram demonstrated a significantly different QRS complex than during tachycardia, both in axis and without the slurred QRS upstroke in leads I and aVL (Figure 2).

The patient was admitted to coronary care unit and was continued on intravenous amiodarone infusion. A subsequent electrophysiological study was performed using single, double and triple extra stimuli from both the right ventricular apex and outflow tract. A non-clinical rapid monomorphic ventricular tachycardia (VT) was induced with double extra stimuli, with resultant hypotension and lightheadedness. Ventricular burst pacing was delivered and converted this VT into the tachycardia with which the patient presented to the hospital. This was clearly ventricular in origin, with AV dissociation. Additional burst pacing terminated this arrhythmia. The patient underwent subsequent placement of a dual-chamber implantable cardioverter-defibrillator.

## Discussion

Tachycardia in an adult is defined as a sustained heart rate above 100 beats per minute. Tachycardia is divided based on QRS complex duration (QRSd) into narrow-complex (QRSd < 120 milliseconds) and wide-complex (QRSd > 120 milliseconds). Narrow complex tachycardia generally utilizes the His-Purkinje system and are thus almost exclusively supraventricular in origin. There are numerous published criteria and algorithms to assist with determining the mechanism of wide-complex tachycardia, though none is perfectly sensitive or specific. The presence of AV dissociation during wide-complex tachycardia, though, is quite specific for ventricular tachycardia.

Cases of narrow-complex tachycardia with AV dissociation have been reported in a young females [1], and a young males [2] without ischemic heart disease. More importantly, there are reports of middle-aged males with a history of myocardial infarction and scarring who presented with a narrow-complex tachycardia which was ventricular in origin [3-4]. These patients also had subtle differences in QRS morphology during tachycardia as compared to sinus rhythm, including axis and precordial transition changes, such as manifested by our patient. It is speculated that these relatively rare cases of narrow-complex ventricular tachycardia utilize the His-Purkinje system as part of the tachycardia circuit [4]. A 1991 study by Hayes, et al. on ventricular tachycardia reported a prevalence of 4.7% (five out of 106) with narrow QRS complexes [5]. These patients were typically misdiagnosed as having supraventricular tachycardia and were treated as such [5]. Compared to the remainder of the cohort, these five patients were younger and had a higher mean ejection fraction [5]. A 2006 study by Bogun, et al. confirmed the involvement of Purkinje fibers in post-infarction ventricular tachycardia which had narrow QRS complexes [6].

In addition to morphologic criteria to help uncover the mechanism of tachycardia, clinical criteria may be very useful. A 1988 study by Tchou, et al., for example, approached the management of wide-complex tachycardia in this manner by asking two simple questions. The first question was whether the patient had ever suffered a myocardial infarction. If affirmative, the second question asked was whether the patient's tachy palpitation problem had started only after the myocardial infarction. If again affirmative, the tachycardia mechanism was ventricular in the vast majority of cases [7]. Applying this simple algorithm to our patient with a borderline QRS duration, the patient would have been promptly diagnosed with ventricular tachycardia.

Our patient had relatively narrow complexes during sustained ventricular tachycardia, likely because the His-Purkinje system constituted a portion of his VT circuit. The relatively narrow QRS complexes led to an initial misdiagnosis of supraventricular tachycardia. Both clinical history and his electrocardiogram variances from his normal QRS complexes supported a mechanism of ventricular tachycardia, which was confirmed by the electrophysiological study. Thus, in patients with coronary artery disease and new-onset tachycardia, even with relatively narrow QRS complexes, a high index of suspicion for VT should be maintained.

## Conclusions

A high index of suspicion should be maintained that any tachycardia, narrow-complex or wide-complex, which commences only after myocardial infarction may be ventricular in origin. Subtle electrocardiogram (EKG) differences during tachycardia versus sinus rhythm should heighten this suspicion. Electrophysiology studies may be useful to help eliminate uncertainties in tachycardia mechanisms in such challenging cases.
